# Supplementary material for: Genetic Variation in CCL18 Gene Influences CCL18 Expression and Correlates with Survival in Idiopathic Pulmonary Fibrosis: Part A
Source: J Clin Med. 2020 Jun 21;9(6):1940. doi: 10.3390/jcm9061940 (PMC7356165; doi:10.3390/jcm9061940)
Supplement: Supplementary file 1 [file jcm-09-01940-s001.pdf]

SUPPLEMENT TO

Genetic variation in CCL18 gene influences CCL18 expression and correlates with survival in Idiopathic pulmonary fibrosis – Part A

by

Ivo A. Wiertz, Sofia A. Moll, Benjamin Seeliger, Nicole P. Barlo, Annette van der Vis, Nicoline M. Korthagen, Ger T. Rijkers, Henk J.T. Ruven, Jan. C. Grutters, Antje Prasse, Coline H.M. van Moorsel

Serum CCL18 analysis

Serum samples of IPF patients were collected at diagnosis. In addition, 100 serum samples of controls were selected for analysis including all controls arrying the minor C allele (n=26) supplemented with a random selection of controls with the rs2015086 TT genotype. Within two hours from blood withdrawal, blood samples were centrifuged for 10 minutes at 2200 rpm, serum was transferred to a new tube and stored at -20°C. Every two months stored samples were moved to –80°C until analysis. CCL18 levels in the derivation cohort were analyzed using a monoplex suspension bead array system. CCL18 antibodies (R&D systems, Minneapolis, MN, USA) were coupled to fluorescent carboxylated beads (Bio-Rad Laboratories, Hercules, CA, USA) according to the manufacturer's protocol (1). Data analysis was performed using the Bioplex 100 system and Bioplex Manager software version 4.1 (Bio-Rad Laboratories, CA, USA). The lower limit of detection was 0.9 pg/ml.

Genotyping of CCL18

In IPF patients, two SNPs with presumed functionality in the promoter region were genotyped (rs712040, rs2015086). In addition, two haplotype tagging SNPs (rs712042, rs712044) were selected to cover genetic variability in the CCL18 gene, using the Tagger program for the genomic region of CCL18 ± 2500 bp on genome build 35. Preferential picking of SNPs was conducted using the pair wise tagging option, a minimum allele frequency setting of 10% and a high Illumina design score. The algorithm was set to select tags that would cover the Caucasian HapMap panel with an r2 of 0.8 or greater (2). DNA was extracted from whole blood samples and SNP typing was conducted using a custom Illumina goldengate bead SNP assay. The assay was performed in accordance with the manufacturer's recommendations (Illumina Inc; San Diego, CA, USA).

RNA expression analysis

PMBCs from healthy donors were isolated from heparinized venous blood using Ficoll-Paque density gradient centrifugation and cryopreserved until further analysis.

Total RNA was isolated from PBMC using de RNeasy microkit (Qiagen, Venlo, The Netherlands) according to the manufacturer's protocol. 0.2 μg RNA was used for first-strand cDNA synthesis using the i-script cDNA synthesis kit (Biorad, Veenendaal, The Netherlands). The obtained cDNA was diluted 1/10 with water of which 4 μl was used for amplification in a reaction volume of 20 μl. Primer sets were purchased from Sigma. The PCR was performed with the RT2 Real-TimeTM SYBR Green PCR master mix (SA-Biosciences, Frederick, USA) according to the manufacturer's protocol. Samples were amplified using a biorad MyiQ real time PCR detection system for 40 cycles (10 sec at 95˚C, 20 sec at 55˚C and 25 sec at 72˚C. The copy number of the CCL18 was normalized by the housekeeping gene β-actin, and is presented as the number of transcripts per 1 copy of β-actin (3).

Supplementary Table S1: characteristics of all IPF patients compared with IPF patients with available serum CCL18.

Characteristics			
***rs2015086* genotype**	**All IPF patients (*n* = 77)**	**IPF with serum CCL18 (*n* = 61)**	***p***
Male, *n* (%)	58 (75)	46 (75)	>0.999
Age, years (IQR)	61.4 (54-72)	61.9 (53-74)	0.796
Former or active smoker, *n* (%)	59 (76)	43 (77)	>0.999
Baseline %FVC predicted, (IQR)	75.7 (62-87)	73.0 (55-87)	>0.999
Baseline %DLCO predicted (IQR)	42.5 (33-56)	41.5 (33-54)	>0.999

IQR (interquartile range); %FVC (Percent of predicted forced vital capacity); %DLCO (Percent of predicted diffusion capacity for carbon monoxide with single breath); p-value calculated by Mann Whitney U test for continuous data and Chi-square test for dichotomous data.

References:

1. de Jager W, Prakken B, Rijkers GT. Cytokine multiplex immunoassay: methodology and (clinical) applications. Methods Mol Biol 2009;514:119-133.
2. de Bakker PI, Yelensky R, Pe'er I, Gabriel SB, Daly MJ, Altshuler D. Efficiency and power in genetic association studies. Nat Genet 2005;37:1217-1223.
3. Thellin O, Zorzi W, Lakaye B, De BB, Coumans B, Hennen G, Grisar T, Igout A, Heinen E. Housekeeping genes as internal standards: use and limits. J Biotechnol 1999;75:291-295.
